# Haemophilus influenzae Pneumonia in an Elderly Couple With Suspected Household Transmission: A Report of Two Cases

**DOI:** 10.7759/cureus.117714

**Published:** 2026-10-09

**Authors:** Moe Kyotani, Tsuneaki Kenzaka, Tomohiro Hayashi

**Affiliations:** 1 Department of Internal Medicine, Hyogo Prefectural Tamba Medical Center, Tamba, JPN; 2 Department of General Medicine, Public Kasumi Hospital, Kami-cho, JPN; 3 Department of Community Medicine (Ibaraki) Graduate School of Medical and Dental Sciences, Institute of Science Tokyo, Tokyo, JPN; 4 Division of Community Medicine and Career Development, Kobe University Graduate School of Medicine, Kobe, JPN

**Keywords:** antimicrobial susceptibility, case report, elderly, haemophilus influenzae, household transmission, pneumonia, respiratory tract infection

## Abstract

*Haemophilus influenzae* is an important cause of community-acquired pneumonia, particularly among older adults and individuals with underlying medical conditions. However, reports of *H. influenzae* pneumonia occurring within a short interval among elderly cohabiting couples with suspected household transmission are limited. Here we present two such cases.

Case 1: A 95-year-old Japanese man presented with a three-day history of productive cough and hoarseness and developed a fever on the day of admission. He had hypoxemia and impaired consciousness. Chest computed tomography (CT) revealed infiltrative opacities, predominantly in the bilateral lower lobes. *Haemophilus influenzae *(*H. influenzae*) was isolated from sputum culture, and his condition improved after 11 days of treatment with ampicillin/sulbactam.

Case 2: A 90-year-old Japanese woman, the wife of the patient in Case 1, developed a productive cough and exertional dyspnea around the time of her husband’s admission. She received oral amoxicillin/clavulanic acid for five days as an outpatient without improvement, and was admitted 10 days after her husband. Chest CT showed multiple infiltrative opacities, predominantly in the right lung, along with diffuse bronchial wall thickening. *Haemophilus influenzae* was isolated from sputum culture, and her condition improved after 12 days of treatment with ceftriaxone.

The *H. influenzae* isolates from the two patients exhibited identical minimum inhibitory concentration profiles for all antimicrobial agents tested. Although strain identity could not be confirmed because molecular epidemiological analyses were not performed, the close temporal relationship between the two illnesses, shared household exposure, and identical antimicrobial susceptibility profiles suggested the possibility of intrahousehold transmission but did not establish strain relatedness or confirm a transmission pathway. These cases provide clinically relevant insights into the possibility of household transmission of *H. influenzae*.

## Introduction

*Haemophilus influenzae* is a major cause of community-acquired pneumonia in adults and an important respiratory pathogen, particularly among older adults and individuals with underlying medical conditions [1,2]. Human-to-human transmission of *H. influenzae *has been documented during outbreaks in extended-care facilities and in studies demonstrating transmission of non-typeable* H. influenzae *(NTHi) between children and their parents [3,4]. However, reports of *H. influenzae* pneumonia occurring within a short interval among elderly couples with suspected household transmission are limited. We report two cases of* H. influenzae* pneumonia in an elderly cohabiting couple, from whom isolates with identical antimicrobial susceptibility profiles were recovered.

## Case presentation

Case 1

A 95-year-old Japanese man with hypertension, dyslipidemia, and hyperuricemia was receiving pravastatin (10 mg/day), candesartan (2 mg/day), icosapent ethyl (1,800 mg/day), and allopurinol (50 mg/day). Three days before admission, he developed hoarseness and a productive cough. He also developed a fever on the day of admission and presented to our hospital.

Upon admission, his Glasgow Coma Scale (GCS) score was E3V5M4, his body temperature was 38.7°C, blood pressure was 105/56 mmHg, pulse rate was 99 beats/min, respiratory rate was 12 breaths/min, and peripheral oxygen saturation (SpO₂) was 85% on room air and 93% with oxygen at 3 L/min. Coarse crackles were heard over the right lung. His heart rhythm was regular, with no apparent murmur.

Laboratory findings (Table 1) included a white blood cell count of 8,630/μL (81.2% neutrophils and 11.0% lymphocytes), blood urea nitrogen of 35.1 mg/dL, creatinine of 1.6 mg/dL, and C-reactive protein (CRP) of 34.3 mg/dL. Chest computed tomography (CT) showed infiltrative opacities, predominantly in the bilateral lower lobes, with a small amount of pleural effusion (Figure 1A). The sputum specimen was of good quality (Geckler classification, group 5). Gram staining showed numerous small, faintly staining, pleomorphic Gram-negative coccobacilli morphologically suggestive of *H. influenzae*, which predominated in the smear; phagocytosis of these organisms by leukocytes was also observed. Two sets of blood cultures were negative, and *H. influenzae* was isolated from the sputum culture. Antimicrobial susceptibility results are shown in Table 2.

**Table 1:** Laboratory data at admission in Case 1 and Case 2 pro-BNP, pro-B-type natriuretic peptide; N/A, not available. The exact assay nomenclature should be confirmed from the laboratory reports.

Parameter	Case 1	Case 2	Reference range
White blood cell count (/μL)	8,630	15,890	4,000-8,000
Neutrophils (%)	81.2	90.4	35.0-80.0
Lymphocytes (%)	11.0	5.2	25.0-55.0
Hemoglobin (g/dL)	12.8	13.2	13.5-17.5
Platelet count (×10⁴/μL)	25.8	33.8	10.0-40.0
Aspartate aminotransferase (U/L)	23	23	13-33
Alanine aminotransferase (U/L)	15	23	8-42
Lactate dehydrogenase (U/L)	208	270	119-229
Alkaline phosphatase (U/L)	384	251	115-359
Total bilirubin (mg/dL)	2.3	1.7	0.2-1.3
pro-BNP (pg/mL)	N/A	11,709	<60
Albumin (g/dL)	2.8	2.6	3.9-4.9
Creatine kinase (U/L)	91	63	62-287
Sodium (mEq/L)	139	141	136-145
Potassium (mEq/L)	4.2	3.1	3.5-5.1
Blood urea nitrogen (mg/dL)	35.1	24.0	8.0-20.0
Creatinine (mg/dL)	1.6	1.0	0.6-1.1
C-reactive protein (mg/dL)	34.3	19.99	<0.50

**Figure 1:**
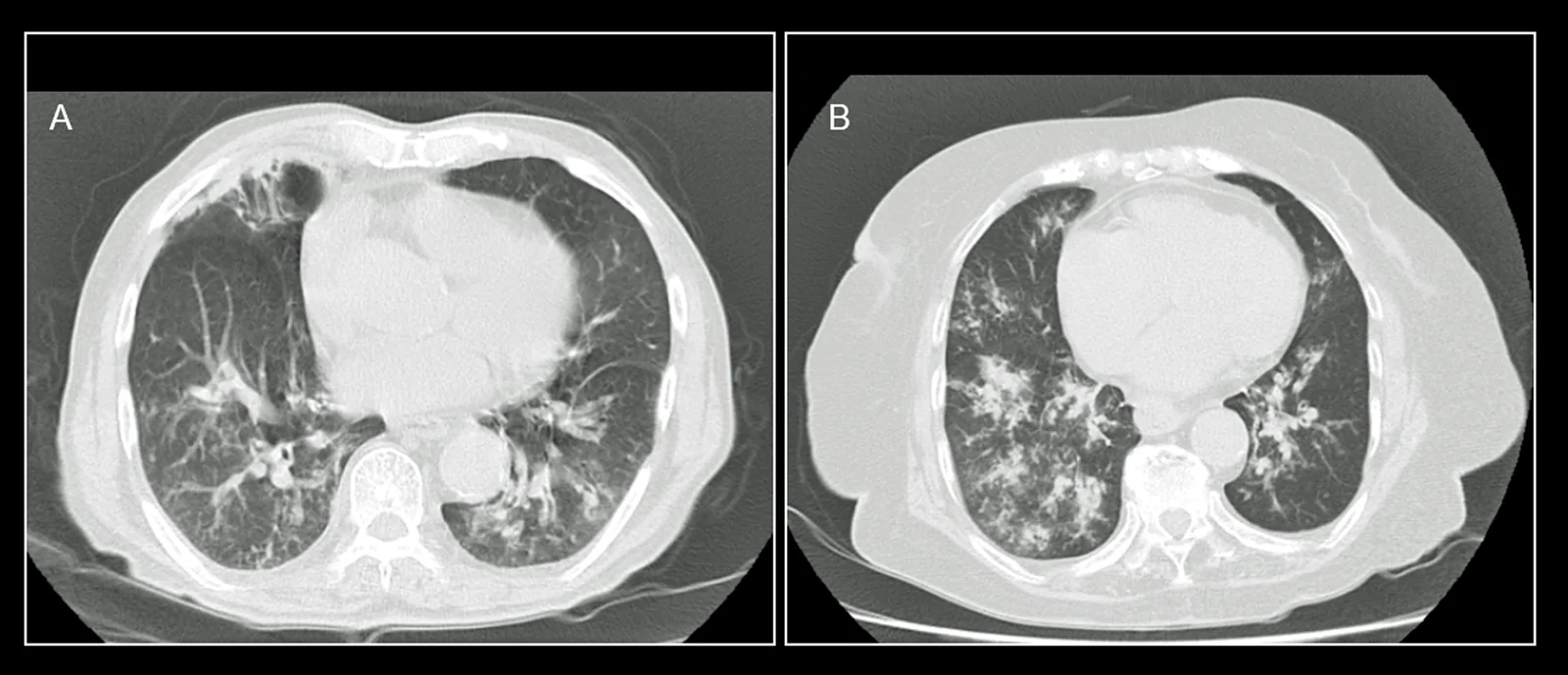
Chest computed tomography (CT) findings at admission. (A) Case 1, a 95-year-old man: bilateral lower-lobe-predominant infiltrative opacities with a small amount of pleural effusion. (B) Case 2, a 90-year-old woman: multiple infiltrative opacities predominantly in the right lung, with diffuse bronchial wall thickening.

**Table 2:** Antibacterial susceptibility of Haemophilus influenzae isolates from sputum cultures in Case 1 and Case 2 MIC, minimum inhibitory concentration. The MIC values for all tested antimicrobial agents were identical between the isolates from Case 1 and Case 2.

Antibacterial agent	Case 1 MIC (μg/mL)	Case 2 MIC (μg/mL)
Ampicillin/sulbactam (ABPC/SBT)	1	1
Ampicillin (ABPC)	>8	>8
Cefepime (CFPM)	<0.25	<0.25
Cefotiam (CTM)	1	1
Ceftriaxone (CTRX)	<0.25	<0.25
Cefditoren (CDTR)	<0.03	<0.03
Meropenem (MEPM)	<0.06	<0.06
Clarithromycin (CAM)	16	16
Ciprofloxacin (CPFX)	<0.25	<0.25
Levofloxacin (LVFX)	<0.25	<0.25
Sulfamethoxazole/trimethoprim (ST)	<4.75	<4.75
Amoxicillin/clavulanic acid (AMPC/CVA)	<1	<1

He was diagnosed with *H. influenzae* pneumonia and was treated with intravenous ampicillin/sulbactam (ABPC/SBT) at 12 g/day. Oxygen therapy was discontinued on hospital day 6, and ABPC/SBT was administered for 11 days. His general condition improved, and he was discharged ambulatory on hospital day 14.

Case 2

A 90-year-old Japanese woman, the wife of the patient in Case 1, had chronic heart failure, atrial fibrillation, and dyslipidemia, and was receiving bisoprolol (1.5 mg/day), furosemide (10 mg/day), dabigatran (220 mg/day), minodronic acid (50 mg/day), ramelteon (8 mg/day), rosuvastatin (2.5 mg/day), azosemide (30 mg/day), and carbocisteine (1,500 mg/day). Around the time her husband was admitted, she developed a productive cough and exertional dyspnea. She received oral amoxicillin/clavulanic acid for five days as an outpatient, without any improvement. She subsequently developed worsening dyspnea, general fatigue, loss of appetite, and difficulty walking, and was admitted 10 days after her husband.

Upon admission, her GCS score was E4V5M6, her body temperature was 36.5°C, blood pressure was 109/54 mmHg, pulse rate was 94 beats/min, respiratory rate was 20 breaths/min, and SpO₂ was 89% on room air and 94% with oxygen at 2 L/min. Coarse crackles and rhonchi were heard bilaterally. Her heart rhythm was irregular, and a systolic murmur was present. Jugular venous distention was noted; however, no lower-extremity edema was observed.

Laboratory findings (Table 1) included a white blood cell count of 15,890/μL (90.4% neutrophils and 5.2% lymphocytes), blood urea nitrogen of 24.0 mg/dL, creatinine of 1.0 mg/dL, CRP of 19.99 mg/dL, and pro-B-type natriuretic peptide (pro-BNP) of 11,709 pg/mL. Chest CT showed multiple infiltrative opacities, predominantly in the right lung, and diffuse bronchial wall thickening (Figure 1B). The sputum specimen was of good quality (Geckler classification, group 5). Gram staining showed numerous small, faintly staining, pleomorphic Gram-negative coccobacilli morphologically suggestive of *H. influenzae*, which predominated in the smear; phagocytosis of these organisms by leukocytes was also observed. *Haemophilus influenzae was *isolated from the sputum culture, and its antimicrobial susceptibility profile was identical to that of the isolate from Case 1 (Table 2).

Although an exacerbation of chronic heart failure was considered because of the markedly elevated pro-BNP level (11,709 pg/mL) and jugular venous distention, chest CT showed no pleural effusion or radiographic evidence of pulmonary congestion. Therefore, exacerbation of chronic heart failure was considered unlikely to be the primary cause of her respiratory deterioration, and acute bronchopneumonia was considered the predominant clinical process.

Given that *H. influenzae* had also been isolated from her husband (Case 1), who had developed pneumonia earlier, her lack of response to outpatient antimicrobial therapy, and her advanced age and underlying medical conditions, intravenous ceftriaxone at 2 g/day was initiated on admission and continued for 12 days. Oxygen therapy was discontinued on hospital day 11. She was able to walk with a cane and was discharged on hospital day 18.

Clinical course of the cohabiting family members

Cases 1 and 2 were a husband and wife who lived in the same household with their 57-year-old son and his 51-year-old wife (Figure 2). Their son developed a sore throat and productive cough 14 days before hospital admission of Case 1. The son’s wife developed a sore throat and productive cough three days before hospital admission of Case 1, and her symptoms improved after treatment with clarithromycin prescribed at a local clinic (Figure 3). No microbiological testing was performed on either family member; therefore, the causative pathogen remained unknown.

**Figure 2:**
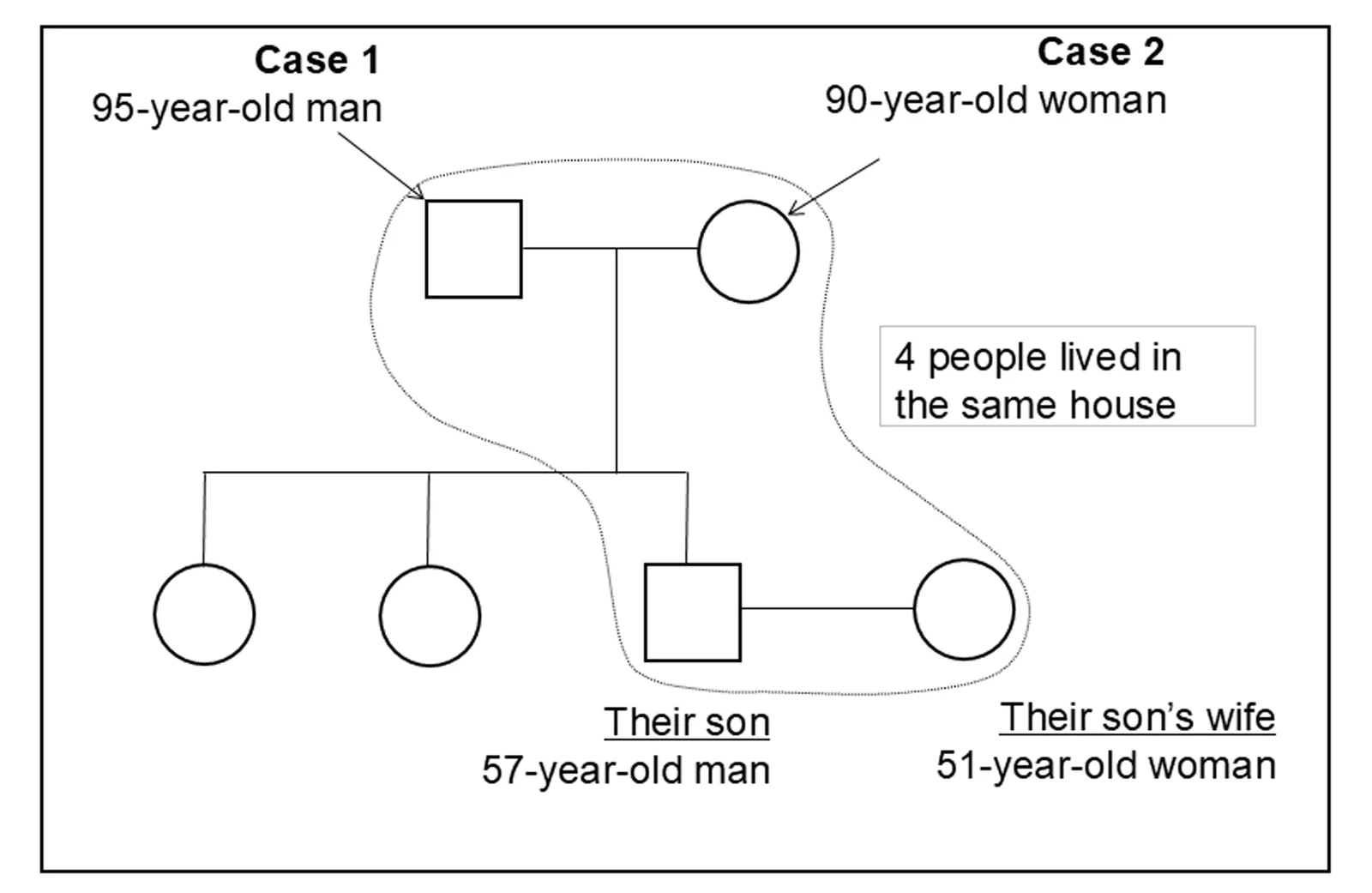
Family tree and household composition. Cases 1 and 2 were husband and wife who lived in the same household with their 57-year-old son and his 51-year-old wife. Squares indicate men and circles indicate women.

**Figure 3:**
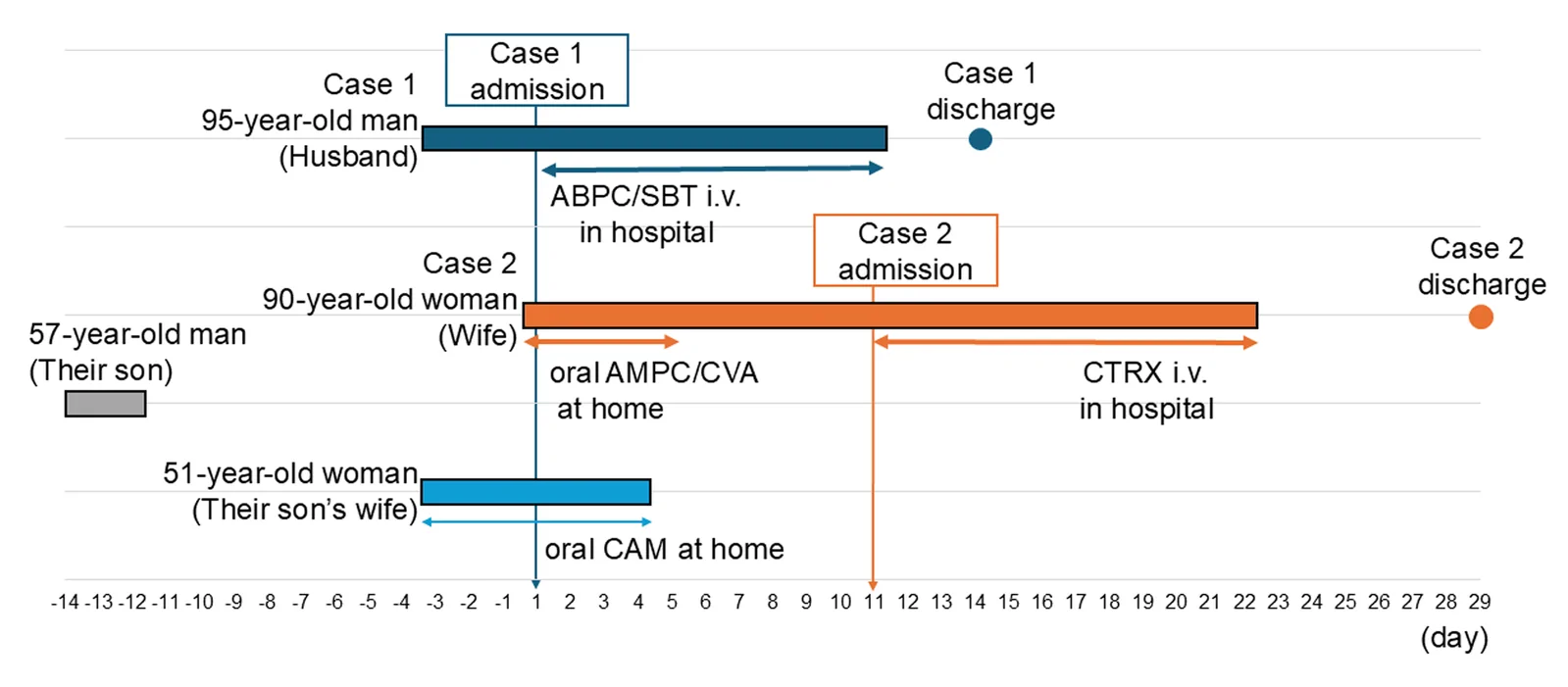
Timeline of respiratory symptoms, hospitalizations, and antimicrobial treatment in the four household members. The day of hospital admission for Case 1 is denoted as Day 1. ABPC/SBT, ampicillin/sulbactam; AMPC/CVA, amoxicillin/clavulanic acid; CAM, clarithromycin; CTRX, ceftriaxone; i.v., intravenous.

## Discussion

A notable feature of these cases is that an elderly cohabiting couple developed *H. influenzae* pneumonia 10 days apart, and that the isolates recovered from their sputum showed identical antimicrobial susceptibility profiles. *Haemophilus influenzae *infection is an important cause of pneumonia in adults, particularly in older adults [1,2]. In invasive* H. influenzae *infections, pneumonia and bacteremia are the major clinical manifestations in adults, and older patients have been reported to have a high prevalence of underlying diseases and an increased risk of death [5]. *Haemophilus influenzae* is classified into encapsulated serotypes a-f and non-encapsulated, non-typeable strains (NTHi), which are particularly important in adult respiratory tract infections [6,7]. Serotyping and capsular typing were not performed in our patients; therefore, we could not determine whether the isolates were NTHi.

Regarding the transmission of* H. influenzae*, an outbreak of non-typeable infections has been reported in an extended-care facility [3]. In addition, a study of 18 child-parent pairs found identical pulsed-field gel electrophoresis (PFGE) patterns in 14 pairs (77.8%), supporting the possibility of household transmission [4]. Molecular epidemiological analyses were not performed in our cases; therefore, isolates from Cases 1 and 2 could not be concluded to represent the same strain. Nevertheless, the close temporal occurrence of pneumonia in a cohabiting couple, the isolation of *H. influenzae* from both patients, and identical minimum inhibitory concentration (MIC) profiles for all tested antimicrobial agents constitute circumstantial evidence consistent with household transmission. However, the possibility of infection from a common external source and by distinct strains with similar susceptibility profiles cannot be excluded.

Furthermore, the couple’s son and daughter-in-law developed upper respiratory symptoms around the onset of illness in Case 1. As sputum culture and molecular testing were not performed for either of them, it is unknown whether their illness was caused by* H. influenzae* or whether they were involved in the transmission chain. Therefore, the direction of transmission within the household could not be determined. The temporal relationship shown in Figure 3 suggests sequential respiratory illnesses within the household but does not establish a transmission pathway.

From the perspective of antimicrobial resistance, an increasing prevalence of β-lactamase-non-producing ampicillin-resistant *H. influenzae* (BLNAR) has been reported in Japan [8-10]. In BLNAR, amino acid substitutions in penicillin-binding protein 3 (PBP3) associated with mutations in the *ftsI* gene contribute to reduced susceptibility to β-lactam antimicrobial agents [9]. Cases of β-lactamase-producing amoxicillin/clavulanate-resistant *H. influenzae* (BLPACR), which combine β-lactamase production with PBP3 alterations, have also been described [11]. Nationwide surveillance has further underscored the importance of continued monitoring of the antimicrobial susceptibility of *H. influenzae *[12]. In the present cases, both isolates had an ampicillin MIC of >8 μg/mL and identical MIC profiles for all other antimicrobial agents tested. However, because results of the β-lactamase testing and *ftsI* gene analysis were unavailable, the isolates could not be classified as BLPAR, BLNAR, or BLPACR on the basis of the available data alone. Thus, the mechanism of resistance cannot be inferred from the microbiological data obtained.

This report has several limitations. First, serotyping, β-lactamase testing, PFGE, multi-locus sequence typing, and whole-genome sequencing were not performed; therefore, strain identity could not be established between the two patients. Second, sputum cultures were not obtained from the other two household members, precluding determination of the direction of transmission within the household. Third, although an acute exacerbation of chronic heart failure was considered in Case 2 because of the markedly elevated pro-BNP level and jugular venous distention, the absence of pleural effusion or pulmonary congestion on chest CT made it unlikely to be the primary cause of her respiratory deterioration; however, a minor contribution from chronic heart failure could not be completely excluded. Due to these limitations, the source and direction of transmission could not be determined. Transmission may have occurred between household members, from a shared source, or the infections may have occurred independently. Therefore, these cases should be regarded as suggestive of possible intrahousehold transmission rather than evidence of a proven transmission pathway.

## Conclusions

Herein, we reported two cases of *H. influenzae* pneumonia that occurred within a short interval in an elderly cohabiting couple, with isolates showing identical antimicrobial susceptibility profiles. Although strain identity and direction of transmission could not be established because molecular epidemiological analyses were not performed, the temporal association and matching antimicrobial susceptibility profiles raised the possibility of intrahousehold transmission but did not establish a transmission pathway. When respiratory infections occur sequentially in households that include older adults or individuals with underlying medical conditions, clinicians should consider the possibility of household transmission and perform appropriate microbiological testing while reinforcing standard infection-control measures.
